# Do Students with Dyslexia Have a Different Personality Profile as Measured with the Big Five?

**DOI:** 10.1371/journal.pone.0064484

**Published:** 2013-05-17

**Authors:** Wim Tops, Ellen Verguts, Maaike Callens, Marc Brysbaert

**Affiliations:** 1 Department of Experimental Psychology, Ghent University, Ghent, Belgium; 2 vzw Kinsbergen Oriëntatiecentrum, Antwerp, Belgium; University of Leicester, United Kingdom

## Abstract

**Background:**

Few studies are available about the personality profile of higher education students with dyslexia and to which extent this could be any different from their non-dyslexic peers.

**Aims and Sample(s):**

To obtain empirical evidence, we compared the personality profile of a group of 100 Dutch-speaking students with dyslexia with that of a control group of 100 students without learning disabilities.

**Methods:**

The NEO-PI-R based on the Big Five in personality research was used.

**Results and Conclusions:**

Our study showed no differences in the personality between both groups. This agrees with a recent meta-analysis of English findings (Swanson & Hsieh, 2009), suggesting that students with dyslexia do not perceive themselves differently than their non-dyslexic peers. Practical implications and directions for future research are considered.

## Introduction

Although reading and writing are mastered well by most persons, they can be a source of frustration for people with dyslexia. This is particularly true during the school years, when good reading and writing skills are important for academic achievement and career building [1]. All beginning readers experience difficulties but for most of them automatization builds up rapidly and after a while they can read effortlessly. This is not true for a group that keeps on having difficulties into adult life, despite normal intelligence, adequate instruction, and often intensive remedial teaching. These persons are confronted with extra challenges compared to their non-dyslexic peers.

Although reading and writing are the core deficits in adults with dyslexia [2]–[3], various authors have suggested that other cognitive skills may be affected as well. Adults with dyslexia have been reported to experience (working) memory problems [4], attentional deficits [5], reduced processing speed [2], problems with fast word finding and arithmetic [6]–[7], and less elaborated vocabulary skills [8]. Dyslexia has also been associated with less sophisticated study strategies and metacognitive skills [9]–[10], higher anxiety, lower self-esteem, problems with coping, and deficient academic achievement and motivation throughout the life-span [11]–[13].

To our knowledge, little is known about the consequences of this adversity on the personality development of students with dyslexia. The only empirical study we could find was Waldo, McIntosh, and Koller [14]. These authors examined the MMPI-derived personality profiles of 165 adults with learning disabilities. Three groups were distinguished on the basis of a comparison of verbal and performance IQ [15]. People with a verbal IQ significantly below their performance IQ were assumed to have a verbal learning disorder; people with the reverse pattern were classified as having a non-verbal learning disorder. These two groups were compared to a control group with similar scores on verbal and performance IQ. No overall differences between the groups were observed, but the two groups with learning disabilities scored higher on some MMPI scales than the control group. The study of Waldo et al. [14] was included in the meta-analysis of Swanson and Hsieh [3], who concluded that there was only a small effect size of d = .28 between the personalities of adults with and without dyslexia.

The MMPI-test is typically used for clinical populations. Therefore, it would be interesting to know whether larger differences can be found on personality tests meant for non-clinical research. The most widely used tests in personality research measure personality traits. A personality trait is a hypothetic attribute that influences a person's behavior, thoughts and feelings across situations. According to the trait approach, differences in personality can be described with a limited set of dimensions. A trait usually is a continuum between two opposed characteristics (e.g., happy versus sad). The approach was introduced in the second half of the twentieth century, when authors like Cattell and Eysenck used factor analysis to examine the pattern of correlations between personality characteristics. Cattell [16] concluded that 16 bipolar traits were needed for a detailed description of one's personality, whereas Eysenck argued that three traits were enough (e.g., [17]). Gradually agreement emerged that five traits represented the optimal number. These five traits became known as the Big Five [18].

The first dimension of the Big Five is *extraversion*. Extraverted people have more attention for and are more oriented towards aspects outside themselves. They are more sociable, more active, and more cheerful. They like excitement and action. They are also strongly focused on their direct environment. In contrast, introverted people are more distant and prefer to be alone. They have a focus on their inside, on their emotions, feelings, and thoughts. The second aspect within the Big Five is *neuroticism*. On the one end of the continuum, there is emotional lability. This is associated with generalized fear and negative feelings such as anger, frustration and shame. On the other end, there are people with high emotional stability who can better dismiss these negative feelings. The third dimension is *openness* to experiences, intellectual or cultural challenges. Open minded people are more (intellectually or culturally) curious and have a rich imagination. They like variation and are able to make independent judgments. There is a positive correlation between this factor and intelligence and educational level. People who lack openness show more conventional behavior and are more conservative. Fourth, *agreeableness* is a measure for interpersonal behavior and orientation to other people's experiences, interests and goals. One gets a high score for agreeableness, if one is cooperative, friendly and helpful. People with low scores tend to be more egocentric and antagonistic. The final dimension of the Big Five is *conscientiousness*. This refers to an individual's conscience as a directive and control mechanism for one's behavior. Expediency, self discipline and thoughtfulness are important facets of this category. Traditionally the Big Five are measured with a personality questionnaire. Two of the most frequently used are the NEO-PI-R [18] and the Five-Factor Personality Inventory [19].

We compared the answers of students with and without dyslexia on a Big Five questionnaire, to see whether the two groups perceive their own personality differently. Each group consisted of 100 participants, so that we could obtain stable estimates of the effect sizes.

## Methods

This study was approved by the ethical comity of Ghent University, meaning that the researchers followed the ethical protocol of the university. All students gave written informed consent and were informed that they could stop at any time if they felt they were treated incorrectly.

### Participants

Two hundred first-year undergraduate students of higher education participated in the study, both non-academic students and university students. One group consisted of 100 students diagnosed with dyslexia, the other was a control group of 100 students with no known neurological or functional deficiencies. All had normal or corrected-to normal vision and were native speakers of Dutch. They all attended higher education in Ghent, one of the major cities of Flanders (the Dutch-speaking northern half of Belgium). Students were paid for their participation.

The students with dyslexia have been thoroughly assessed [2], [20] and meet the three criteria for dyslexia as outlined by the SDN [21]. The SDN uses a descriptive definition of dyslexia. In their guidelines dyslexia is defined as an impairment characterized by a persistent problem in learning to read and/or write words or in the automatization of reading and writing. First, the level of reading and/or writing of the students with dyslexia was significantly lower than what could be expected on the basis of their educational level and age. All students with dyslexia had (sub) clinical scores (<pc 10) on a word reading test (*EMT* [One Minute Test] [22]) and/or, pseudo word reading test (*De Klepel* [23]) and/or word spelling test (GL&SCHR [24]). Secondly, most students had low scores despite taking some form of remedial teaching, which meant they met the criterion “resistance to instruction” [25]. Finally, the SDN definition requires ensuring that the reading and writing impairment cannot be attributed to external and/or individual factors such as socio-economic status, cultural background or intelligence, which was the case for the group we examined.

A group of 100 control students was recruited matched on age, gender and field of study, using the social networks of the students, student coaches and electronic learning platforms. None of the members of the control group had known neurological or functional disorders.

There was no difference between the two groups in socio-economical level based on the educational level of the mother [χ^2^(3) = 4.855, p = .183] and father [χ^2^(3) = 2.634, p = .452]. Educational levels were: lower secondary education, higher secondary education, postsecondary education either at university or non-university college. Both groups had slightly above average fluid intelligence and did not differ from each other. Table 1 shows the main characteristics of the groups (see [2] for more details).

**Table 1:** General Information About the Student Groups With and Without Dyslexia.

		students without dyslexia N M (*SD*)		students with dyslexia N M (*SD*)		Effect size Cohen's *d*
Gender	Male	46		46		
	Female	54		54		
Studies	University	66		66		
	College for higher education	34		34		
Age		19.40	*(1.00)*	19.11	*(0.70)*	NA
Fluid IQ		106.80	*(10.80)*	105.40	*(11.00)*	−0.13
Word reading		100.40	*(10.60)*	77.00	*(14.20)*	−1.97
Pseudoword reading		59.70	*(13.10)*	40.90	*(10.50)*	−1.59
Word spelling		24.60	*(2.80)*	17.50	*(4.00)*	−2.05

*Note*. TIQ = Total IQ score (KAIT; Dekker, Dekker, & Mulder, 2004); Word reading = Dutch word reading, number of words read correctly in 1 minute time (EMT; Brus & Voeten, 1991); Pseudoword reading = number of pseudowords read correctly in 1 minute time (de Klepel; van den Bos et al., 1999); word spelling = number of words spelled correctly in a word dictation task (GL& De Pessemier & andries, 2009). Effect sizes calculated according to Cohen's *d* (positive d-values represent better performance of the controls and negative values better performance of the students with dyslexia).

### Test

We administered a computerized Dutch version of the NEO-PI-R [18], [26]. This is a self-report questionnaire comprising 30 facet scales (based on 240 items) that can be reduced to the five dimensions of the Five Factor Model. Administration took 40 to 50 minutes on average. Because half of the participants had reading problems, no time constraints were imposed.

### Procedure

The personality test was part of a larger protocol about dyslexia in higher education [2]. The complete test battery involved additional tests such as an intelligence test, reading and spelling tests, and a structured interview about the functioning and the well-being of the student. It was administered in two sessions of about three hours each. The protocol was divided into two counterbalanced parts. The order of tests in part one and two was fixed and chosen to avoid succession of similar tests. The two parts were counterbalanced (i.e., half of each group started with part one, the other half with part two).

For the personality test, students were seated in front of a computer screen in a well-lit and quiet room. The test administrator was present, but could not see the computer screen, nor the answers the students gave. If necessary, students could ask to have a question read aloud or explained to them. The latter usually involved the explanation of an unfamiliar word, for which the test administrator gave a synonym or a meaningful context. Students could also ask for breaks if necessary. If more than 40 answers (on a total of 240) were left open, the participant's results were excluded for further analysis. This was the case for one student from the dyslexia group. Other missing answers were replaced by the mean, neutral score. This was done in less than 0.01% of the questions for both groups.

## Results

For many practical purposes, statistical significance is secondary to effect size, because statistical significance depends on the sizes of the groups tested as much as on the difference between the groups. Therefore, all results are additionally given as effect sizes derived from parametric t- tests (conclusions were not different when we used a non-parametric Mann-Whitney U-test). These are calculated as follows: 
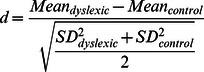
Values of t are not given, as these can easily be calculated from the d-scores:
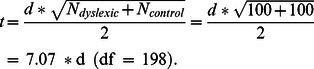



The sign of the d-values was adapted so that positive d-values always represented better performance of the controls and negative values better performance of the students with dyslexia. Because we had two groups of 100 participants, the 95% confidence intervals of the obtained d-scores are ±.4, (calculated with ESCI-CI delta [27]). Effect sizes smaller than d = .4 do not exceed the normal variability observed within the population much and are often considered not to be of practical relevance.

As can be seen in Table 2, there was only one effect size above.4: Students with dyslexia reported that they were less sensation seeking than the controls. All other differences were smaller than.3. The largest effect size was observed for neuroticism (d = .26) in the advantage of the students with dyslexia (meaning that they reported lower levels of neuroticism) but without practical relevance.

**Table 2:** Results for NEO-PI-R for Both Student Groups Expressed as Effect Sizes (Cohen's d).

	Students with dyslexia		Students without dyslexia			
	M	*(SD)*	M	*(SD)*	d	
***Neuroticism***	**147.180**	***(19.77)***	**150.76**	***(19.44)***	**−0.26**	
Anxiety	25.77	*(5.09)*	26.30	*(4.89)*	−0.15	
Angry hostility	21.86	*(4.67)*	22.76	*(4.10)*	−0.30	
Depression	25.08	*(5.14)*	26.17	*(5.43)*	−0.29	
Self consciousness	24.82	*(4.68)*	24.96	*(4.59)*	−0.04	
Impulsiveness	28.07	*(4.34)*	28.87	*(3.69)*	−0.28	
Vulnerability	21.66	*(4.33)*	21.68	*(4.20)*	−0.01	
***Extraversion***	**165.23**	***(18.66)***	**167.88**	***(19.81)***	**0.20**	
Warmth	29.49	*(4.36)*	29.54	*(4.29)*	0.02	
Gregariousness	28.18	*(5.039)*	28.81	*(4.66)*	0.18	
Assertiveness	24.04	*(4.368)*	23.79	*(5.25)*	−0.07	
Activity	26.03	*(3.84)*	25.80	*(4.21)*	−0.08	
Excitement seeking	27.70	*(4.10)*	29.20	*(4.12)*	0.52	*
Positive emotion	29.79	*(4.33)*	30.50	*(4.611*	0.22	
***Openness***	**167.85**	***(16.64)***	**168.97**	***(17.45)***	**0.10**	
Fantasy	29.58	*(5.26)*	29.60	*(4.57)*	0.01	
Aesthetics	27.30	*(7.21)*	27.61	*(5.44)*	0.07	
Feelings	29.140	*(4.50)*	30.06	*(4.00)*	0.31	
Actions	23.81	*(3.92)*	24.33	*(4.74)*	0.17	
Ideas	27.29	*(5.01)*	27.34	*(5.448)*	0.02	
Values	29.76	*(3.38)*	29.90	*(3.90)*	0.06	
***Agreeableness***	**165.69**	***(19.55)***	**165.44**	***(14.74)***	**−0.02**	
Trust	28.47	*(4.65)*	27.65	*(4.43)*	−0.26	
Straightforwardness	27.05	*(5.27)*	27.24	*(4.65)*	0.05	
Altruism	29.86	*(3.47)*	30.33	*(3.24)*	0.20	
Compliance	23.49	*(4.38)*	22.93	*(4.02)*	−0.19	
Modesty	27.99	*(4.59)*	27.80	*(3.98)*	−0.06	
Tender mindedness	28.72	*(4.00)*	29.47	*(3.58)*	0.28	
***Conscientiousness***	**152.35**	***(20.29)***	**150.36**	***(20.52)***	**−0.14**	
Competence	26.62	*(3.16)*	26.29	*(3.92)*	−0.13	
Order	23.22	*(5.02)*	23.19	*(5.09)*	−0.01	
Dutifulness	28.14	*(3.89)*	28.24	*(4.35)*	0.03	
Achievement striving	27.35	*(4.07)*	26.57	*(4.77)*	−0.25	
Self discipline	24.19	*(4.81)*	23.39	*(4.98)*	−0.23	
Deliberation	22.97	*(5.41)*	22.79	*(5.22)*	−0.05	

*Note*. * p<.05.

Because gender is known to have impact on personality scores [28], we also ran separate analyses for the male and female participants. As can be seen in Tables 3 and 4, the lack of a difference in personality profile between students with dyslexia and controls was true for both genders.

**Table 3:** Results for NEO-PI-R for the Male Students Expressed as Effect Sizes (Cohen's d).

	Men with dyslexia		Men without dyslexia		
	M	*(SD)*	M	*(SD)*	d
**Neuroticism**	**141.45**	***−20.93***	**144.10**	***−19.72***	**−0.09**
Anxiety	23.98	−*5.31*	24.27	−*4.79*	0.04
Angry hostility	22.00	−*5.10*	22.59	−*4.25*	0.09
Depression	23.55	−*4.99*	24.88	−*5.64*	0.18
Self consciousness	23.83	−*4.55*	23.61	−*4.58*	−0.04
Impulsiveness	28.00	−*3.76*	28.59	−*3.72*	0.11
Vulnerability	20.05	−*4.33*	20.17	−*4.24*	0.02
**Extraversion**	**163.48**	−***20.28***	**164.02**	**−** ***22.73***	**0.02**
Warmth	27.76	−*4.62*	27.73	−*4.12*	−0.01
Gregariousness	27.36	−*5.65*	27.66	−*5.07*	0.04
Assertiveness	24.79	−*4.01*	23.54	−*5.36*	−0.19
Activity	26.38	−*4.29*	25.57	−*4.65*	−0.13
Excitement seeking	28.17	−*4.12*	29.88	−*4.30*	0.29
Positive emotion	29.02	−*4.62*	29.66	−*4.95*	0.09
**Openness**	**166.14**	**−** ***15.49***	**164.78**	**−** ***15.78***	**−0.06**
Fantasy	30.38	−*4.79*	29.61	−*4.06*	−0.12
Aesthetics	26.62	−*5.36*	25.46	−*5.53*	−0.15
Feelings	27.76	−*4.33*	28.56	−*3.90*	0.14
Actions	23.41	−*4.23*	23.83	−*4.76*	0.07
Ideas	28.62	−*4.71*	28.29	−*5.90*	−0.04
Values	29.35	−*3.48*	28.79	−*3.72*	−0.11
**Agreeableness**	**156.57**	**−** ***19.62***	**159.95**	**−** ***14.85***	**0.14**
Trust	27.60	−*5.31*	26.83	−*5.03*	−0.11
Straightforwardness	24.57	−*5.21*	25.95	−*4.65*	0.20
Altruism	28.41	−*3.47*	29.17	−*3.06*	0.17
Compliance	22.79	−*4.16*	23.02	−*3.66*	0.04
Modesty	26.10	−*4.96*	26.78	−*4.26*	0.11
Tender mindedness	27.12	−*4.26*	28.39	−*3.23*	0.24
**Conscientiousness**	**148.71**	**−** ***19.10***	**148.78**	**−** ***21.08***	**0.00**
Competence	26.83	−*3.01*	26.92	−*4.25*	0.02
Order	21.64	−*4.64*	22.83	−*4.18*	0.19
Dutifulness	27.50	−*4.20*	27.41	−*4.58*	−0.01
Achievement striving	26.81	−*4.16*	25.78	−*4.73*	−0.16
Self discipline	23.19	−*4.61*	22.83	−*5.11*	−0.05
Deliberation	22.93	−*5.31*	23.00	−*5.53*	0.01

**Table 4:** Results for NEO-PI-R for the Female Students Expressed as Effect Sizes (Cohen's d).

	Women with dyslexia		Women without dyslexia		
	M	*(SD)*	M	*(SD)*	d
**Neuroticism**	**151.33**	**−** ***17.94***	**155.39**	**−** ***17.99***	**−0.16**
Anxiety	27.07	−*4.54*	27.71	−*4.48*	−0.10
Angry hostility	21.76	−*4.37*	22.91	−*4.03*	−0.19
Depression	26.19	−*5.00*	27.12	−*5.10*	−0.13
Self consciousness	25.53	−*4.69*	25.89	−*4.40*	−0.06
Impulsiveness	28.12	−*4.84*	29.07	−*3.68*	−0.16
Vulnerability	22.83	−*3.97*	22.73	−*3.87*	0.02
**Extraversion**	**166.50**	**−** ***17.46***	**170.56**	**−** ***17.21***	**0.17**
Warmth	30.75	−*3.72*	30.80	−*3.97*	0.01
Gregariousness	28.78	−*4.50*	29.60	−*4.22*	0.13
Assertiveness	23.50	−*4.57*	23.97	−*5.21*	0.07
Activity	25.78	−*3.49*	25.97	−*3.90*	0.04
Excitement seeking	27.36	−*4.09*	28.73	−*3.97*	0.24
Positive emotion	30.35	−*4.06*	31.09	−*4.30*	0.13
**Openness**	**169.09**	**−** ***17.46***	**171.88**	**−** ***18.08***	**0.11**
Fantasy	29.01	−*5.54*	29.59	−*4.93*	0.08
Aesthetics	29.47	−*5.21*	29.10	−*4.89*	−0.05
Feelings	30.14	−*4.39*	31.10	−*3.76*	0.17
Actions	24.10	−*3.69*	24.68	−*4.73*	0.10
Ideas	26.33	−*5.03*	26.69	−*5.05*	0.05
Values	30.06	−*3.31*	30.68	−*3.86*	0.12
**Agreeableness**	**172.29**	**−** ***16.77***	**169.25**	**−** ***13.52***	**−0.14**
Trust	29.10	−*4.04*	28.22	−*3.90*	−0.16
Straightforwardness	28.85	−*4.57*	28.14	−*4.47*	−0.11
Altruism	30.91	−*3.08*	31.14	−*3.14*	0.05
Compliance	24.00	−*4.50*	22.86	−*4.27*	−0.18
Modesty	29.36	−*3.77*	28.51	−*3.65*	−0.16
Tender mindedness	29.88	−*3.40*	30.22	−*3.65*	0.07
**Conscientiousness**	**154.98**	**−** ***20.88***	**151.46**	**−** ***20.23***	**−0.12**
Competence	26.47	−*3.28*	25.86	−*3.65*	−0.12
Order	24.36	−*5.01*	23.44	−*5.66*	−0.12
Dutifulness	28.60	−*3.61*	28.81	−*4.12*	0.04
Achievement striving	27.75	−*3.99*	27.12	−*4.76*	−0.10
Self discipline	24.91	−*4.85*	23.78	−*4.89*	−0.17
Deliberation	23.00	−*5.54*	22.64	−*5.04*	−0.05

## Discussion

Students with dyslexia in higher education above all are confronted with serious reading and spelling impairments [3], [5]. This was also true for the group we tested [2], [20]. Reading and writing skills were the most impaired, resulting in effect sizes around d = 2.0. As for other cognitive skills, such as verbal and visual memory, problem solving and abstract reasoning, students with dyslexia differed much less from their non-dyslexic peers. All these factors resulted in small to moderate effect sizes [2].

Within the dyslexia literature there is, however, a debate about whether dyslexia is an isolated reading and/or writing deficit or a broader deficit affecting multiple cognitive, metacognitive, and socio-emotional domains, which may affect the personality. For instance, Mason and Mason [29] argued that students with learning disabilities frequently experience low self-esteem, which has negative consequences for their emotional state and well-being. The authors even claimed that because of the adversity people with dyslexia are confronted with the social dimensions of life can be bigger challenges for them than for their peers without reading and writing deficits. Unfortunately, this debate is not based on a rich set of empirical data. The few studies that exist were summarized by Swanson and Hsieh [3] as part of a wide-range meta-analysis. They reported only a small difference in personality between participants with and without dyslexia, but the data were mainly based on studies with the MMPI, a test mostly used for clinical populations. We addressed the shortage of data by examining the answers of two large groups on a widely used Big Five personality questionnaire.

Like the previous studies, summarized in Swanson and Hsieh [3], we too failed to find big differences between students with and without dyslexia (Tables 2, 3, 4). Furthermore, the small differences we found were often in favor of the students with dyslexia, who had better scores on neuroticism, agreeableness, and conscientiousness. Only for extraversion and openness did they score slightly lower (Table 2). The fact that this pattern was not exactly the same for males and females (Tables 3 and 4) testifies to the small sizes of the differences.

The similarity of the personality profiles of students with and without dyslexia goes against the claims made by some authors about the consequences of dyslexia (see above). The fact that the extra challenges students with dyslexia are faced with do not affect their scores on personality tests, is more in line with the view of personality as a stable construct over time and across circumstances [30], rather than with a view of personality as moldable and influenced by specific experiences [31].

Two comments should be made about our findings, however. The first is that our data are based on self-reports. So, a more valid summary of our findings might be that students in higher education do not perceive their own personality differently than control students. Theoretically, it is possible that students with dyslexia differ from those without, but do not notice it themselves. This possibility is made less plausible, however, by the observation of Swanson and Hsieh [3] that students with dyslexia are perceived similarly by third parties as controls. If anything, according to the meta-analysis third parties have a slightly more positive relationship with dyslexic students than with controls, a finding in agreement with our data.

The second comment to be made about our findings is that they pertain to a group of students with dyslexia, who despite all adversity have been reasonably successful. After all, they have managed to get into higher education. It is possible that the more negative personality consequences of dyslexia prevent other people from reaching this potential. This is a limitation that should be kept in mind when interpreting our findings. On the other hand, there are two factors mitigating this interpretation. The first is that in Belgium all students are allowed to enter higher education when they successfully completed secondary education. This is different from the Anglo-Saxon system in which institutes of higher education are allowed to impose extra entrance requirements such as SAT-scores or A-levels. Because of the lack of entrance requirements, the first year of higher education in Belgium is considered as a selection year, with only half of the students expected to complete the study they started. So, the students we tested were not sure of successful completion of their study (which by itself can be considered a potential source of stress). The second mitigating factor is that two thirds of the students we tested did not go to university but to a college of higher education providing a so-called professional bachelor directly applied to specific professions. Given that a university degree by many is considered as the ideal to strive for, not all of our students may have perceived themselves as fully successful.

All in all, we believe that our empirical data (together with those of Swanson and Hsieh [3]) give a quite realistic picture of the personality of students who come to higher education with an assessment (or suspicion) of dyslexia. These students seem to have more resilience to deal with the extra challenges they are confronted with than the doom scenarios sometimes portrayed. At the same time, we agree that our findings are limited to those students who start studying in higher education. Only a prospective, longitudinal study can inform us about the implications of dyslexia (and other learning difficulties) on personality for the full range of abilities.
