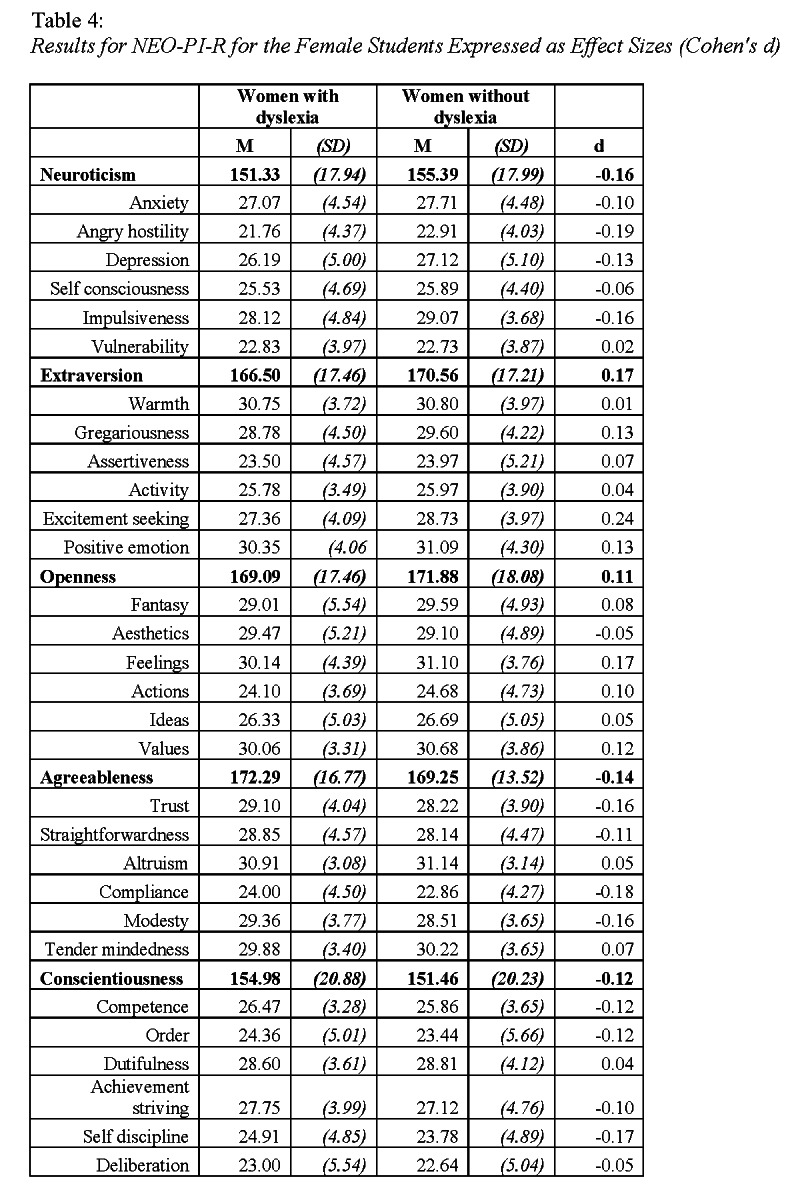# Correction: Do Students with Dyslexia Have a Different Personality Profile as Measured with the Big Five?

**DOI:** 10.1371/annotation/65014249-b018-4d08-9def-90c049d5e41b

**Published:** 2013-05-21

**Authors:** Wim Tops, Ellen Verguts, Maaike Callens, Marc Brysbaert

In Table 3 and Table 4, the values for standard deviations were incorrectly listed as negative values.

The correct Table 3 can be viewed here: 

**Figure:**
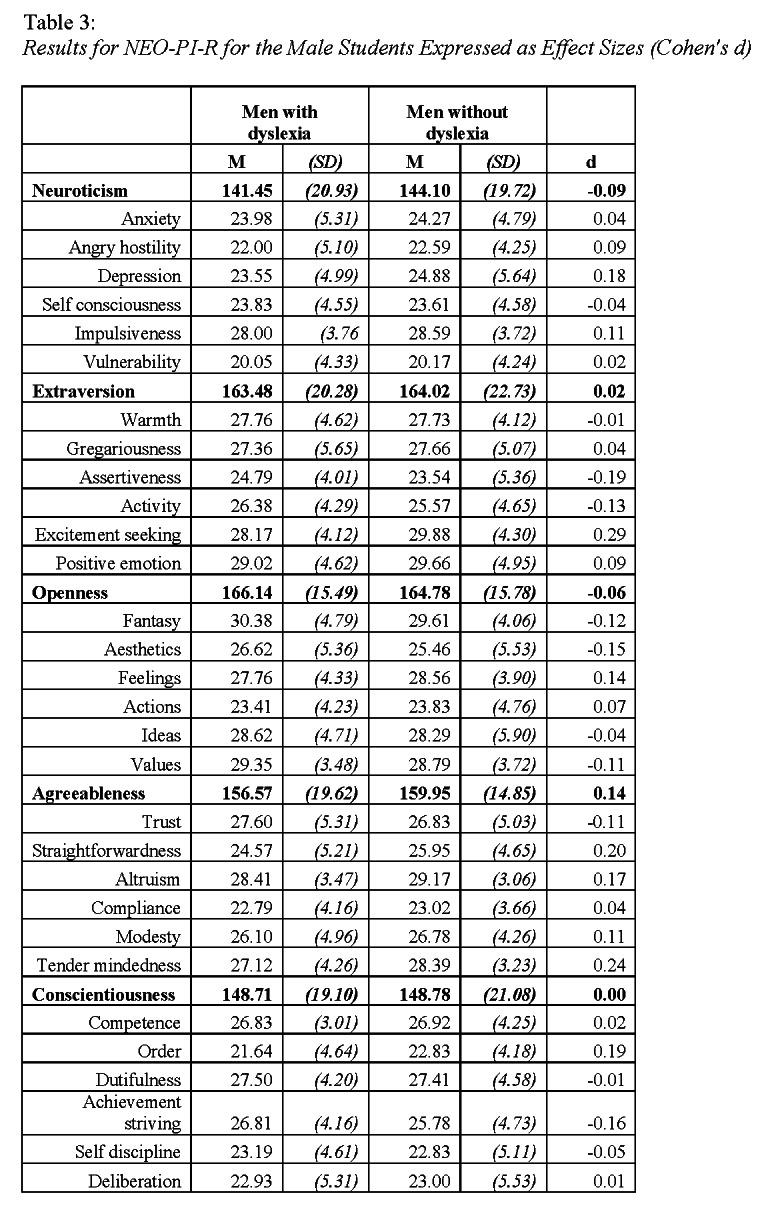


The correct Table 4 can be viewed here: 

**Figure:**